# Phase I EnACT Trial of the Safety and Tolerability of a Novel Oral Formulation of Amphotericin B

**DOI:** 10.1128/AAC.00838-20

**Published:** 2020-09-21

**Authors:** Caleb P. Skipper, Mucunguzi Atukunda, Anna Stadelman, Nicole W. Engen, Ananta S. Bangdiwala, Katherine H. Hullsiek, Mahsa Abassi, Joshua Rhein, Melanie R. Nicol, Eva Laker, Darlisha A. Williams, Raphael Mannino, Theresa Matkovits, David B. Meya, David R. Boulware

**Affiliations:** a Infectious Diseases Institute, Makerere University, Kampala, Uganda; b University of Minnesota, Minneapolis, Minnesota, USA; c Matinas Biopharma, Bedminster, New Jersey, USA; d College of Health Sciences, Makerere University, Kampala, Uganda

**Keywords:** *Cryptococcus*, amphotericin B, pharmacokinetics, HIV, cryptococcal meningitis, antifungal agents, human immunodeficiency virus

## Abstract

Amphotericin B deoxycholate (AMB) has substantial toxicities. A novel encochleated amphotericin B deoxycholate (cAMB) formulation has oral bioavailability, efficacy in an animal model, and minimal toxicity due to targeted drug delivery into macrophages, where intracellular fungi reside. We conducted a phase I, ascending-dose trial of cAMB administered at 1.0 g, 1.5 g, or 2.0 g per day in 4 to 6 divided doses among HIV-positive survivors of cryptococcosis (*n* = 9 per cohort). We assessed the tolerability of cAMB and the adverse events (AEs) associated with cAMB treatment over 3 days. A second trial (*n* = 9) assessed the tolerability of 1.5 g/day given for 7 days. In the single-ascending-dose study, all subjects received their full daily dose without vomiting (100% tolerability). The cohort receiving 1.0 g had 4 transient clinical AEs in 2 subjects within 48 h and 8 laboratory AEs (*n* = 6 grade 2, *n* = 2 grade 1). The cohort receiving 1.5 g had 7 clinical AEs in 1 subject attributed to acute gastroenteritis (*n* = 4 grade 2) and 5 laboratory AEs (*n* = 1 grade 2). The cohort receiving 2.0 g had 20 clinical AEs among 5 subjects within 48 h (*n* = 3 grade 2) and 11 laboratory AEs (*n* = 2 grade 2, *n* = 1 grade 3). From a qualitative survey, 26 of 27 subjects (96%) preferred their experience with oral cAMB over their prior experience with intravenous (i.v.) AMB. The second, multiple-dose cohort received 1.5 g/day for 1 week, with 98.4% (248/252) of the doses being taken. Overall, 5 clinical AEs (*n* = 5 grade 1) and 6 laboratory AEs (*n* = 6 grade 1) occurred without kidney toxicity. Oral cAMB was well tolerated when given in 4 to 6 divided daily doses without the toxicities commonly seen with i.v. AMB. (This study has been registered at ClinicalTrials.gov under registration no. NCT04031833.)

## Abstract (precis)

Amphotericin B deoxycholate (AMB) has substantial toxicities. A novel encochleated amphotericin B deoxycholate (cAMB) formulation has oral bioavailability, efficacy in an animal model, and minimal toxicity due to targeted drug delivery into macrophages, where intracellular fungi reside. We conducted a phase I, ascending-dose trial of cAMB administered at 1.0 g, 1.5 g, or 2.0 g per day in 4 to 6 divided doses among HIV-positive survivors of cryptococcosis (*n* = 9 per cohort).

## INTRODUCTION

Intravenously (i.v.) administered amphotericin B deoxycholate (AMB) is traditionally considered the vanguard antifungal therapy for serious fungal infections due to its broad activity and low resistance rates. However, i.v. AMB has numerous significant toxicities, including anemia, nephrotoxicity, and infusion-related reactions. Further, i.v. AMB has historically been available only as an intravenous medication, as oral absorption in humans is limited and causes toxicity (1). Encochleated amphotericin B deoxycholate (cAMB; Matinas Biopharma Holdings, Bedminster, NJ) is a novel lipid nanocrystal formulation designed for targeted oral delivery of the classically intravenously administered antifungal.

**FIG 1:**
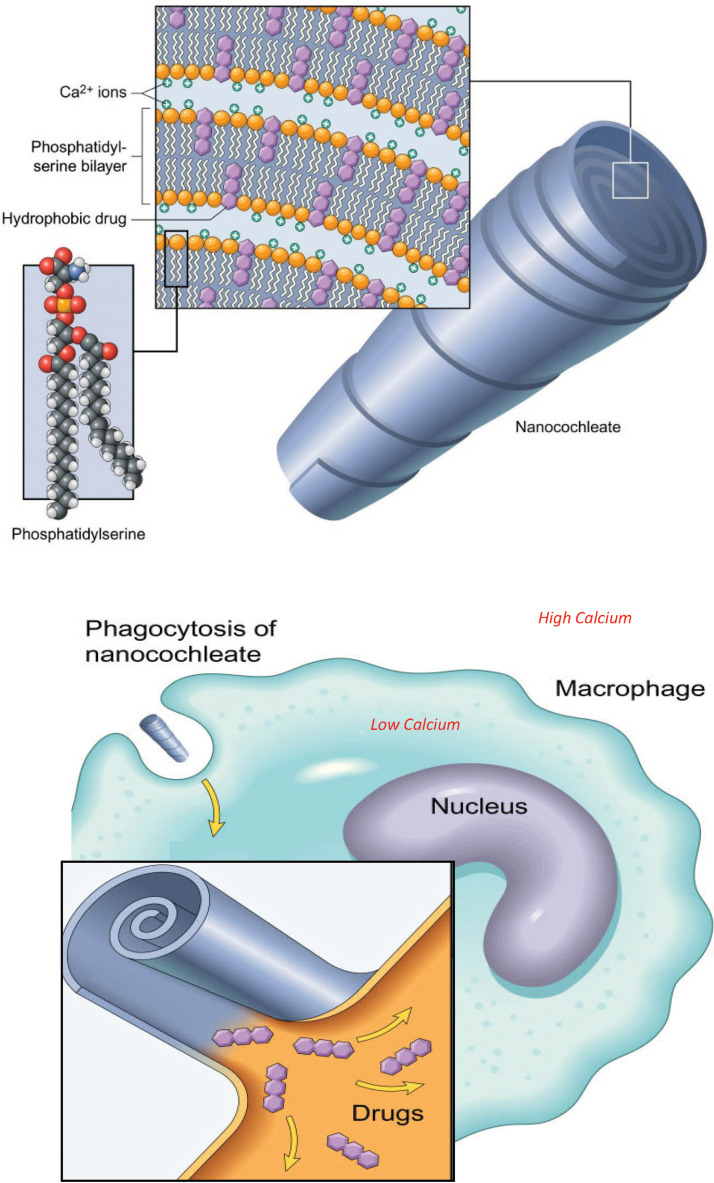
Illustration of cochleate technology. Amphotericin B is embedded within the phosphatidylserine bilayers of the cochleate, along with calcium ions. The cochleate protects the cAMB from digestive processes in the gastrointestinal tract until absorbed. Once absorbed, the cochleate is phagocytosed by macrophages. Due to the higher calcium concentration in the cochleate than in the cytoplasm of the macrophage, the cochleate is pulled open as calcium ions rush out, allowing intracellular delivery of the amphotericin B.

The cAMB cochleates are comprised of a solid lipid bilayer sheet rolled into a spiral in which amphotericin molecules are trapped, along with calcium (Fig. 1). The drug is made biologically available when the cochleate is taken up by a phagocytic cell and a gradient between the high calcium concentration in the cochleate and the lower cytoplasmic calcium concentration is created. This results in the cochleate opening and releasing the amphotericin molecules. The cochleate structure provides protection against degradation in unfavorable environments (i.e., low pH in the stomach) while allowing targeted intracellular delivery into macrophages and reticuloendothelial cells. The high intracellular concentrations allow for killing of intracellular organisms (such as fungi), while the low plasma concentrations reduce systemic toxicities.

cAMB demonstrates *in vitro* activity against Cryptococcus neoformans, *Candida* spp., and Aspergillus fumigatus (Matinas investigator brochure). In a cryptococcosis mouse model experiment by Williamson and colleagues, mice given cAMB at 25 mg/kg of body weight plus flucytosine (5FC) had rates of survival equivalent to those of mice injected with AMB plus 5FC (2). Further, these animal experiments demonstrated that cAMB is colocalized with cryptococcal yeast inside macrophages in the brain. Human data are also available for cAMB, including data from two unpublished noncryptococcosis clinical trials. One trial assessing the safety and efficacy of cAMB in vulvovaginal candidiasis (Clinical Trials.gov registration no. NCT02971007) demonstrated excellent safety at 400 mg/day with no serious adverse events (SAEs), although microbiological cure was suboptimal compared to that achieved with fluconazole. The second trial (Clinical Trials.gov registration no. NCT02629419) is ongoing in a small number of persons with azole-resistant severe chronic mucocutaneous candidiasis related to a primary immune deficiency. All these participants had a positive clinical response with symptom improvement scores and were able to take cAMB doses ranging from 200 to 800 mg for an extended duration, at least 18 months. In general, cAMB was well tolerated over a dose range of 200 mg to 800 mg among clinical study participants, with no evidence of hepatotoxicity, nephrotoxicity, or hypokalemia being detected. As single doses increase above 400 mg, gastrointestinal side effects (e.g., nausea, abdominal pain, bloating, diarrhea) occurred more frequently, likely due to unabsorbed cAMB.

Given the animal model efficacy and human safety data demonstrated in previous studies, cAMB is a promising therapeutic option for the treatment of invasive fungal infections, yet the optimal or maximum tolerated dose is unknown. We therefore designed an open-label phase I safety, tolerability, and dose-finding study of cAMB in HIV-positive Ugandans with a history of cryptococcal meningitis. Here, we present the results of participant tolerability and adverse events (AE) recorded in different dosing cohorts. Based on our phase I data, we plan to move cAMB to a phase II efficacy trial for HIV-associated cryptococcal meningitis.

## RESULTS

In the initial single-ascending-dose trial, a total of 35 HIV-positive survivors of cryptococcal meningitis were screened. We enrolled 9 participants into each of the three sequential dosing cohorts: 1.0 g, 1.5 g, and 2.0 g per day. All participants were survivors of prior cryptococcal meningitis (median time since prior diagnosis, 46 months) and had previously received i.v. AMB for ∼14 days. The median age was 36 years (interquartile range [IQR], 30 to 44 years), with 41% (11/27) of the participants being women. All participants were currently receiving antiretroviral therapy (ART), with 15 (42%) taking atazanavir and 37% (10/27) actively taking fluconazole at 200 mg as prophylaxis. The baseline characteristics are summarized in Table 1.

**TABLE 1:** Baseline characteristics^*a*^

Characteristic	Phase IA			Phase IB (1.5 g)
	Cohort 1 (1.0 g)	Cohort 2 (1.5 g)	Cohort 3 (2.0 g)	
Demographic				
Median (IQR) age (yr)	33 (30, 44)	39 (30, 43)	42 (35, 44)	34 (31, 40)
No. (%) of female participants	4 (44.4)	2 (22.2)	5 (55.6)	5 (55.6)
Median (IQR) ht (cm)	165.8 (158.2, 168.5)	165.7 (163.3, 167.2)	166.0 (153.2, 168.8)	164.2 (156.4, 165.8)
Median (IQR) wt (kg)	63.5 (60.5, 68.5)	57.5 (55.5, 68.0)	75.0 (65.5, 78.0)	69.5 (61.5, 79.8)
Median (IQR) value for the following vital signs:				
Systolic BP (mm Hg)	108 (104, 112)	124 (120, 132)	112 (107, 125)	127 (113, 136)
Diastolic BP (mm Hg)	70 (60, 72)	79 (73, 88)	73 (68, 83)	78 (69, 82)
Pulse (bpm)	69 (64, 74)	70 (69, 79)	65 (60, 78)	74 (66, 83)
HIV infection history				
No. (%) of HIV-positive participants	9 (100.0)	9 (100.0)	9 (100.0)	9 (100.0)
No. (%) of participants currently on ART	9 (100.0)	9 (100.0)	9 (100.0)	9 (100.0)
Median (IQR) time (mo) on ART	41.9 (10.7, 43.2)	28.6 (16.1, 52.4)	42.0 (19.7, 47.0)	41.4 (18.6, 44.6)
CM history				
No. (%) of participants with previous blood CrAg screen	9 (100.0)	9 (100.0)	9 (100.0)	9 (100.0)
No. (%) of participants with history of CM	9 (100.0)	9 (100.0)	9 (100.0)	9 (100.0)
Median (IQR) time (mo) since last diagnosis	45.3 (44.3, 46.2)	48.1 (41.4, 51.9)	49.1 (44.7, 51.1)	48.5 (47.8, 50.6)
No. (%) of participants with prior i.v. AMB therapy	9 (100.0)	9 (100.0)	9 (100.0)	9 (100.0)
No. (%) of participants with other medical conditions/medications				
Currently with another medical condition	1 (11.1)	2 (22.2)	0 (0.0)	0 (0.0)
Currently taking fluconazole	2 (22.2)	5 (55.6)	3 (33.3)	2 (22.2)
Currently taking co-trimoxazole	3 (33.3)	1 (11.1)	3 (33.3)	2 (22.2)

a Nine participants were enrolled in each cohort. Abbreviations: BP, blood pressure; ART, antiretroviral therapy; bpm, numbers of beats per minute; CrAg, cryptococcal antigen; CM, cryptococcal meningitis.

To meet the tolerability criteria, the participants were required to take the full daily dose without vomiting within 30 min after taking any dose, to report AEs of grade 2 or less, and to not discontinue the cAMB due to an AE or participant choice. For the cohorts receiving 1.0 g and 1.5 g, all 18 participants met the full tolerability criteria. In the cohort receiving 2.0 g, all 9 participants completed the dosing, but 1 participant experienced a grade 3 laboratory AE of transient thrombocytopenia at 48 h of unclear etiology. Therefore, only 89% (8/9) of the participants met the full tolerability criteria with dosing at 2.0 g.

Of the 27 participants in the phase IA study, 8 (30%) experienced at least one clinical AE. A total of 31 unique clinical AEs were reported, with 24 (77%) being grade 1 and 7 (23%) being grade 2. Increasing numbers of AEs correlated with increasing cAMB dose, with 65% (20/31) being recorded in the cohort receiving 2.0 g. No grade 3, grade 4, or serious AEs occurred. The most common clinical AEs were abdominal pain (*n* = 5), nausea (*n* = 5), and dizziness (*n* = 4). Of the same 27 participants, 15 (56%) experienced at least one laboratory AE. A total of 24 laboratory AEs were reported, with 14 (58%) being grade 1 and 9 (38%) being grade 2. There was one grade 3 adverse event, in which a participant in the cohort receiving 2.0 g had platelet counts of 163,000 and 160,000 cells/μl at baseline and 24 h, respectively, which then dropped to 31,000 cells/μl at 48 h. No platelet clumping was reported. The platelet count was repeated several times to ensure AE resolution; the count rebounded to 172,000 cells/μl by 2 weeks. The participant had no clinical deterioration or evidence of bleeding at the time of the documented thrombocytopenia. No grade 4 or serious laboratory AEs occurred. The most common laboratory AEs were an elevation in the total bilirubin level (*n* = 6), a mild isolated elevation in the aspartate transaminase (AST) level (*n* = 5), and mild sodium abnormalities (*n* = 2 hyponatremic; *n* = 3 hypernatremic) which met the National Institute of Allergy and Infectious Diseases (NIAID) Division of AIDS (DAIDS) criteria for a grade 1 AE (146 to 149 meq/liter) but which was within the normal range of the local College of American Pathologists (CAP)-certified laboratory for the population (138 to 150 meq/liter). Of the subjects with elevations in AST levels, only one was accompanied by an elevation in the alanine transaminase level, which occurred in a subject with chronic hepatitis B. The AE prevalence is summarized in Table 2.

**TABLE 2:** Adverse event summary

Characteristic	Phase IA			Phase IB (1.5 g)
	Cohort 1 (1.0 g)	Cohort 2 (1.5 g)	Cohort 3 (2.0 g)	
No. of participants	9	9	9	9
No. of participants with SAEs	0	0	0	0
No. of participants with unexpected events	0	0	0	0
No. (%) of clinical events	4	7	20	5
Grade 1	4 (100)	3 (43)	17 (85)	5 (100)
Grade 2	0	4 (57)	3 (15)	0
Grade 3	0	0	0	0
Grade 4	0	0	0	0
No. (%) of laboratory events	8	5	11	6
Grade 1	2 (25)	4 (80)	8 (73)	6 (100)
Grade 2	6 (75)	1 (20)	2 (18)	0
Grade 3	0	0	1 (9)	0
Grade 4	0	0	0	0

A dose of 1.5 g was selected as the 100% tolerated phase IA study dose for use in phase IB of the study. The demographics of the 9 phase IB participants were similar to those of the phase IA participants. Eight of the 9 participants completed full dosing through 7 days, with 1 participant unintentionally missing 1 day of dosing. Three participants experienced a total of five grade 1 clinical AEs, with none being serious. Three participants also experienced a total of six grade 1 laboratory AEs, and again, none met the criteria for a serious AE. Overall, the participants from phase IB took cAMB successfully as outpatients and displayed minimal evidence of intolerance or toxicity with 7 days of continuous therapy.

**FIG 2:**
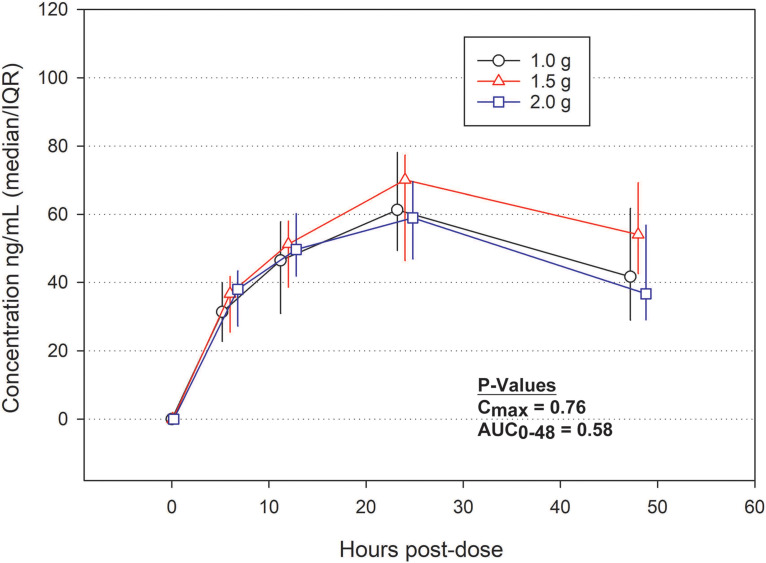
Pharmacokinetic data from the EnACT (encochleated oral amphotericin for cryptococcal meningitis) phase IA study. The median AUCs from time zero to the last quantifiable concentration in the plasma of the cohorts receiving doses of 1.0 g, 1.5 g, and 2.0 g were 1,970 ng · h/ml (IQR, 1,660 to 2,480 ng · h/ml), 2,660 ng · h/ml (IQR, 1,940 to 2,910 ng · h/ml), and 2,180 ng · h/ml (IQR, 1,790 to 2,630 ng · h/ml), respectively. The median cAMB plasma concentrations at various time points are visually displayed in the line graph. No statistically significant differences in *C*_max_ or AUC were found between groups. Error bars represent the interquartile range for each cohort. The *P* values were calculated by the Kruskal-Wallis ANOVA. Abbreviations: AUC_0-48_, area under the curve from 0 to 48 h; *C*_max_, maximum concentration in plasma.

The plasma concentrations of cAMB were measured at 0, 6, 12, 24, and 48 h from the time of cAMB initiation, and the area under the curve (AUC) was calculated. Interestingly, the plasma AUC from 0 to 48 h (AUC_0–48_) did not significantly differ across the three dosing cohorts, with the overall median being 2,200 ng · h/ml (*P* = 0.58) (Fig. 2). Based on elimination between the 24-h and 48-h time points, the median half-life of cAMB was 48 h, and the median maximum concentration in plasma (*C*_max_) averaged across the three cohorts was 59.2 ng/ml (IQR, 48.8 to 74.0 ng/ml). In pharmacokinetic analyses of the phase IB cohort, the plasma concentration at 24 h (74.8 ng/ml) was 77% of the day 7 concentration of 97.7 ng/ml. At 72 h, the plasma concentration of 91.1 ng/ml was 93% of the day 7 concentration.

Using a qualitative scale, we assessed the impressions of the participants in the phase IA cohort of their experience with cAMB compared with their prior experience with i.v. AMB. In the preparticipation survey, 40% (10/25) indicated that they would be willing to receive i.v. AMB again for their cryptococcal meningitis versus an alternative therapy. After completing the study follow-up, 96% (26/27) of the participants indicated the they would prefer cAMB to i.v. AMB for cryptococcal meningitis treatment. Overall, 93% (25/27) thought that the multiple doses of cAMB were very convenient for therapy, whereas 65% (15/23) of the respondents thought that i.v. AMB was very convenient for therapy. One participant could not recall the initial i.v. AMB therapy. Nausea severity scoring was rated as “none” by 8 of 9 participants in the 1-g and 1.5-g cohorts and 7 of 9 participants in the 2.0-g cohort. In comparison, 25% (6/24) recalled severe nausea with i.v. AMB.

## DISCUSSION

We found that cAMB administered in 4 to 6 divided daily doses was generally safe and tolerated in healthy HIV-positive Ugandans at doses of up to 2.0 g/day. While mild AEs were more frequently encountered as the dose was increased, 95% (34/36) of the participants completed all prescribed doses without a grade 3 or worse AE. It is worth noting that we found that 2017 DAIDS laboratory AE criteria occasionally graded a laboratory value (i.e., the sodium level) as a grade 1 event when the number fell within the reference range of the Ugandan population studied at our CAP-certified laboratory. Therefore, some of our grade 1 events may have been overreported. Based on our data, 1.5 g daily may represent the sweet spot of optimal tolerability versus dosing escalation.

cAMB appears to have a favorable toxicity profile compared to that of i.v. AMB. Data taken from the 177-participant COAT (cryptococcal optimal ART timing) trial (3) recorded 23 (13%) grade 3 to 5 AEs involving low serum potassium levels and 18 (10%) grade 3 to 5 AEs involving high serum creatinine levels, with all participants having received i.v. AMB. In comparison, our cAMB participants had no grade 3 or higher AEs involving potassium or creatinine, even when dosed for 7 days. Similar to the findings of previous unpublished studies (Matinas investigator brochure), our phase I data suggest that cAMB has much less electrolyte and kidney toxicity than i.v. AMB.

We found that smaller, frequent dosing helped mitigate the gastrointestinal side effects seen in early studies that utilized larger, one-time dosing. For example, a prior phase I study (CAM-102) of healthy volunteers given cAMB at 800 mg daily in a single dose reported that 58% (7/12) of participants developed a gastrointestinal AE (Matinas investigator brochure), whereas only 11% (2/18) of our participants developed any gastrointestinal AEs, despite receiving 1,000 and 1,500 mg daily with dosing divided 4 and 5 times daily, respectively. Further, our participants overwhelmingly declared that their subjective experience with cAMB was better than that with i.v. AMB.

We also demonstrated improved absorption and plasma concentrations of cAMB with smaller, frequent doses in phase I study volunteers. Given as a single 800-mg dose, the median AUC from 0 to 24 h (AUC_0–24_) was 636 ng · h/ml and the mean *C*_max_ was 40.8 ng/ml (an ∼7% increase compared to the *C*_max_ of 38.1 ng/ml achieved after a single 400-mg dose) (Matinas investigator brochure). In contrast, giving 1,000 mg in 4 to 6 divided doses increased the AUC_0–24_ to 990 ng · h/ml and the *C*_max_ to 64.9 ng/ml (∼60% more than that after a single 800-mg dose). Interestingly, we saw negligible differences in *C*_max_ or AUC between our cohorts receiving divided doses of 1.0 g, 1.5 g, and 2.0 g (Fig. 2). This is hypothesized to be secondary to the targeted intracellular delivery of the drug and minimal drug in the plasma compartment, despite the improved absorption with divided dosing. Alternatively, there may have been a saturation of absorption, limiting increases in plasma concentrations; however, the current hypothesis based on data from animal studies is that plasma concentrations correlate poorly with antifungal activity, as increased oral cAMB dosing results in greater antifungal activity (4). We do note that cAMB plasma concentrations are an order of magnitude lower than the *C*_max_ reported with i.v. AMB in humans (1,430 ng/ml at a single 0.6-mg/kg dose) (5); thus, the pharmacokinetics of cAMB do not directly compare to those of i.v. AMB. The efficacy of cAMB in human fungal disease remains unknown, and our phase II trial will provide important refinements in the pharmacokinetic/pharmacodynamic correlates for the treatment of cryptococcal meningitis with cAMB.

Our successful phase I cAMB trial does have limitations. We used survivors of prior cryptococcal meningitis for the advantage of the subjective comparison of cAMB to i.v. AMB; however, these participants may not have reported mild AEs as frequently, given their historical perspective of illness and daily concurrent antiretroviral and antibiotic use. In our pharmacokinetic analysis, the reported half-life represents an observed value, given our sampling time points, rather than the true elimination half-life.

Per our data safety monitoring board (DSMB) review, we are moving forward with a phase II randomized trial evaluating the efficacy of cAMB in HIV-positive Ugandans with cryptococcal meningitis using early fungicidal activity as the primary endpoint. Persons will be given stage-based incremental decreases in the i.v. AMB duration while concurrently increasing the cAMB duration in order to determine cAMB efficacy while prioritizing participant safety. Based on our phase I data, we plan to use cAMB at 2.0 g daily with flucytosine for cryptococcal meningitis induction therapy, followed by cAMB at 1.5 g with fluconazole for 4 weeks of consolidation therapy. Persons with acute cryptococcal meningitis and elevated intracranial pressure commonly have significant nausea and vomiting, and therefore, the tolerability will be an ongoing area of monitoring. However, 2-week cryptococcal meningitis survivors discharged home with improving symptoms may find 1.5 g to be more tolerable for the additional 4 weeks of therapy. We began the phase 2 EnACT (encochleated oral amphotericin for cryptococcal meningitis) trial enrollment in July 2020. cAMB remains a promising novel antifungal in the fight against serious fungal infections, including cryptococcosis.

## MATERIALS AND METHODS

We conducted a phase I safety and tolerability trial for cAMB in HIV-positive Ugandans from October 2019 to January 2020 in Kampala, Uganda (ClinicalTrials.gov registration no. NCT04031833). The trial was designed to occur in two sequential phases, with 27 participants enrolled in phase IA and 9 participants enrolled in phase IB. The participants in phase IA received a single ascending dose of cAMB sequentially administered at 1.0 g, 1.5 g, and 2.0 g per day in 4 to 6 divided doses, with 9 participants being used in each dose group. At least 6 of 9 participants had to tolerate the dose before proceeding to the next dosing cohort. Dose-limiting intolerability was defined as experiencing a grade 3 or higher AE, vomiting within 30 min after taking a dose, or discontinuing the medication due to an AE or toxicity. The participants in phase IB received the cAMB dose that was 100% tolerated in phase IA for 7 days to verify safety and tolerability with continuous dosing. Participants in phases IA and IB had the same inclusion and exclusion criteria.

Participants were recruited from a prior cryptococcal meningitis trial cohort (ASTRO-CM [adjunctive sertraline for the treatment of HIV-associated cryptococcal meningitis]; ClinicalTrials.gov registration no. NCT01802385) (6). All recruited subjects had a history of resolved HIV-associated cryptococcal meningitis and had received i.v. AMB previously. Inclusion requirements were an age of >18 years and a calculated creatinine clearance of >70 ml/min/1.73 m^2^ (measured within 3 months). Participants were excluded if they exhibited a current illness, had a known untreated significant health problem, or had received amphotericin B therapy in the past 90 days. Women who were pregnant or breast-feeding were excluded.

Trial endpoints included (i) the proportion of the daily dose received and tolerated without vomiting within 30 min; (ii) the cumulative score of nausea, vomiting, and diarrhea by Common Terminology Criteria AE (CTCAE)-graded criteria (7); (iii) the incidence of adverse events (AEs) by the National Institute of Allergy and Infectious Diseases (NIAID) Division of AIDS (DAIDS) toxicity scale (version 2017) for grade 1 to 5 or serious AEs (SAEs) (8); and (iv) the participant subjective impression(s) of cAMB by visual analog and other scales. All clinical and laboratory AEs were followed up until resolution.

We collected plasma for pharmacokinetic analyses at 0, 6, 12, 24, and 48 h in phase IA. We monitored complete blood count, chemistries, and kidney and liver function tests at 0, 24, and 48 h, with follow-up occurring thereafter for any abnormal results. For phase IB, the same laboratory monitoring occurred at +1 day, +3 days, and +7 days for safety assessment and pharmacokinetic analyses. All laboratory measurements were performed at the IDI Core Laboratory, which is a College of American Pathologists (CAP)-certified laboratory and which participates in external quality assurance for all laboratory assays. Plasma drug quantification was conducted at Medpace Bioanalytical Laboratories (Medpace Inc., Cincinnati, OH) using an internally validated assay (MBL 17301). Briefly, 100 μl of plasma was placed in a 2-ml tube and fortified with internal standard working solution. The analyte and internal standard were extracted via protein precipitation using acetonitrile. The supernatant was then dried under a stream of nitrogen, and the resulting residue was reconstituted in 150 μl of 0.1% formic acid in methanol-water (1:1) before being submitted to liquid chromatography-tandem mass spectrometry analysis on a Sciex Qtrap or Triple Quad mass spectrometer.

Statistical analyses were primarily descriptive. The median and interquartile ranges or the number and proportions are reported, where appropriate. Area-under-the-curve (AUC) and half-life calculations were made with Phoenix WinNonlin (version 8.2) software (Certara, St. Louis, MO), using a linear-up/logarithm-down method, with comparisons for determination of statistical significance being performed by the Kruskal-Wallis analysis of variance (ANOVA) in SAS (version 9.4) software (SAS Institute, Cary, NC). A visual analog scale was used to assess the participants’ subjective experience with various aspects of the trial. The numbers of clinical and laboratory adverse events (AE) were summarized by the Division of AIDS (DAIDS) toxicity table, version 2017.

The Mulago Hospital Research and Ethics Committee, the Ugandan National Council of Science and Technology, the Uganda National Drug Authority, and the University of Minnesota Institutional Review Board approved the study. The study was performed under U.S. Food and Drug Administration (FDA) Investigational New Drug (IND) application number 072807. All participants provided informed consent in their preferred language. An internal review was performed after the study with each dosing cohort to ensure tolerability. A data safety monitoring board (DSMB) reviewed all safety data after completion of phase I data collection.

## References

[1] Halde C, Newcomer VD, Wright ET, Sternberg TH 1957 An evaluation of amphotericin B in vitro and in vivo in mice against Coccidioides immitis and Candida albicans, and preliminary observations concerning the administration of amphotericin B to man. J Invest Dermatol 28:217–231. doi:10.1038/jid.1957.26. 13429161

[2] Lu R, Hollingsworth C, Qiu J, Wang A, Hughes E, Xin X, Konrath KM, Elsegeiny W, Park YD, Atakulu L, Craft JC, Tramont EC, Mannino R, Williamson PR 2019 Efficacy of oral encochleated amphotericin B in a mouse model of cryptococcal meningoencephalitis. mBio 10:e00724-19. doi:10.1128/mBio.00724-19. 31138748 PMC6538785

[3] Boulware DR, Meya DB, Muzoora C, Rolfes MA, Huppler Hullsiek K, Musubire A, Taseera K, Nabeta HW, Schutz C, Williams DA, Rajasingham R, Rhein J, Thienemann F, Lo MW, Nielsen K, Bergemann TL, Kambugu A, Manabe YC, Janoff EN, Bohjanen PR, Meintjes G, COAT Trial Team. 2014 Timing of antiretroviral therapy after diagnosis of cryptococcal meningitis. N Engl J Med 370:2487–2498. doi:10.1056/NEJMoa1312884. 24963568 PMC4127879

[4] Delmas G, Park S, Chen ZW, Tan F, Kashiwazaki R, Zarif L, Perlin DS 2002 Efficacy of orally delivered cochleates containing amphotericin B in a murine model of aspergillosis. Antimicrob Agents Chemother 46:2704–2707. doi:10.1128/aac.46.8.2704-2707.2002. 12121962 PMC127382

[5] Bekersky I, Fielding RM, Dressler DE, Lee JW, Buell DN, Walsh TJ 2002 Pharmacokinetics, excretion, and mass balance of liposomal amphotericin B (AmBisome) and amphotericin B deoxycholate in humans. Antimicrob Agents Chemother 46:828–833. doi:10.1128/AAC.46.3.828-833.2002. 11850268 PMC127462

[6] Rhein J, Huppler Hullsiek K, Tugume L, Nuwagira E, Mpoza E, Evans EE, Kiggundu R, Pastick KA, Ssebambulidde K, Akampurira A, Williams DA, Bangdiwala AS, Abassi M, Musubire AK, Nicol MR, Muzoora C, Meya DB, Boulware DR, ASTRO-CM Team. 2019 Adjunctive sertraline for HIV-associated cryptococcal meningitis: a randomised, placebo-controlled, double-blind phase 3 trial. Lancet Infect Dis 19:843–851. doi:10.1016/S1473-3099(19)30127-6. 31345462 PMC7041360

[7] U.S. Department of Health and Human Services, National Institutes of Health, National Cancer Institute. 2017 Common terminology criteria for adverse events (CTCAE) v5.0. U.S. Department of Health and Human Services, National Institutes of Health, National Cancer Institute, Bethesda, MD.

[8] U.S. Department of Health and Human Services National Institutes of Health, National Institute of Allergy and Infectious Diseases, Division of AIDS. 2017 Division of AIDS (DAIDS) table for grading the severity of adult and pediatric adverse events, corrected version 2.1. U.S. Department of Health and Human Services, National Institutes of Health, National Cancer Institute, Bethesda, MD.

